# *Vibrio cholerae* non-O1 - the first reported case of keratitis in a healthy patient

**DOI:** 10.1186/s12879-019-4475-4

**Published:** 2019-10-29

**Authors:** Wei-Dar Chen, Li-Ju Lai, Wei-Hsiu Hsu, Tsung-Yu Huang

**Affiliations:** 1 Department of Ophthalmology, Chang Gung Memorial Hospital, Chiayi, Taiwan; 2 Department of Chinese Medicine, School of Medicine, Chang Gung University, Taoyuan, Taiwan; 3 Department of Orthopedics, Chang Gung Memorial Hospital, Chiayi, Taiwan; 4 Division of Infectious Diseases, Department of Internal Medicine, Chang Gung Memorial Hospital, Chiayi, Taiwan; 5 Graduate Institute of Clinical Medical Sciences, College of Medicine, Chang Gung University, Taoyuan, Taiwan

**Keywords:** *Vibrio cholerae* non-O1, Keratitis, Virulence factor, Marine shrimp, Corneal ulcer

## Abstract

**Background:**

*Vibrio cholerae* non-O1 is a virulent pathogen that causes significant morbidity and mortality in humans. Herein, we report a case of corneal ulcer caused by this pathogen.

**Case presentation:**

A 59-year-old fisherman with no systemic history was struck in the right eye by a marine shrimp and developed keratitis. Corneal scrapping culture revealed the presence of the *V. cholerae* non-O1, and its identification was confirmed by Analytical Profile Index 20E system and polymerase chain reaction. He was successfully treated with topical levofloxacin (0.3%) and fortified amikacin (12.5 mg/mL) for 2 weeks. The visual acuity recovered to 20/25 after treatment without complications.

**Conclusions:**

This is the first case report of keratitis caused by *V. cholerae* non-O1 strain. Ocular injury by marine creatures and contaminated seawater can contribute to severe corneal ulcer. Early diagnosis can be achieved by meticulous history taking and a comprehensive laboratory workup. Simultaneously, an effective antibiotic therapy can lead to a positive outcome.

## Introduction

*Vibrio cholerae* is a facultative anaerobic gram-negative comma-shaped bacillus that exists ubiquitously in marine and estuarine environments. Exposure to contaminated water and ingestion of raw seafood are crucial routes of infection that causes overwhelming morbidity and mortality in humans [1]. *V. cholerae* serogroup O1 and O139 primarily contribute to pandemic Vibrio gastroenteritis by means of cardinal virulence factors of cholera toxin (CT) and toxin-coregulated pilus (TCP). Similarly, non-cholera Vibrio including *Vibrio parahaemolyticus* and *Vibrio vulnificus* (*V. vulnificus*) can lead to cholera-like diarrhea, open wound infection, necrotizing fasciitis and septicemia via multiple virulence determinants such as capsular polysaccharide, hemolysin, siderophores and metalloproteases [2, 3]. These major pathological *Vibrio* species have been extensively studied for their detrimental effect on public health. However, pestilent *V. cholerae* non-O1/ non-O139 infection has gained attention over the past decade. A few studies have reported cases of extra-intestinal *Vibrio* infections caused by *V. cholerae* non-O1/non-O139 stains, including soft tissue infection, pneumonia, acute cholecystitis, liver abscess, peritonitis, urinary tract infection, septicemia and meningitis in addition to acute enteric illness [4, 5]. Nevertheless, the incidence of ocular infection caused by *V. cholerae* non-O1/non-O139 was rather low. Only one case of endophthalmitis attributed to *V. cholerae* non-O1/non-O139 strain in a cirrhotic patient with septicemia has been reported [6]. Herein, we reported a case of a healthy patient with *V. cholerae* non-O1 keratitis and searched the related literature to discuss pathogenesis, diagnosis and management of *V. cholerae* keratitis.

## Case presentation

**Fig. 1:**
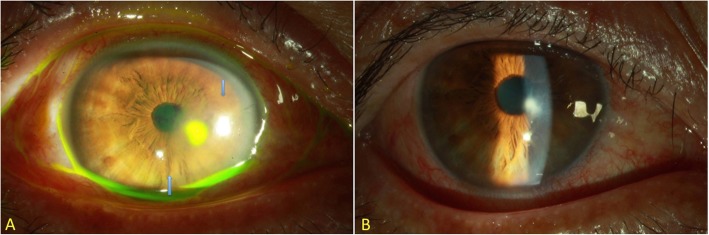
*Vibrio cholerae* (*V. cholerae*) non-O1 keratitis. **a**. Cornea epithelial defect (1.3 × 1.8mm^2^) with dense stromal infiltration on the center cornea. Cornea edema with Descemet membrane folding was also found (5.0 × 5.6mm^2^). Arrows indicated the area of cornea edema. **b** Small cornea opacity without epithelial defect was noted 1 week after treatment

**Fig. 2:**
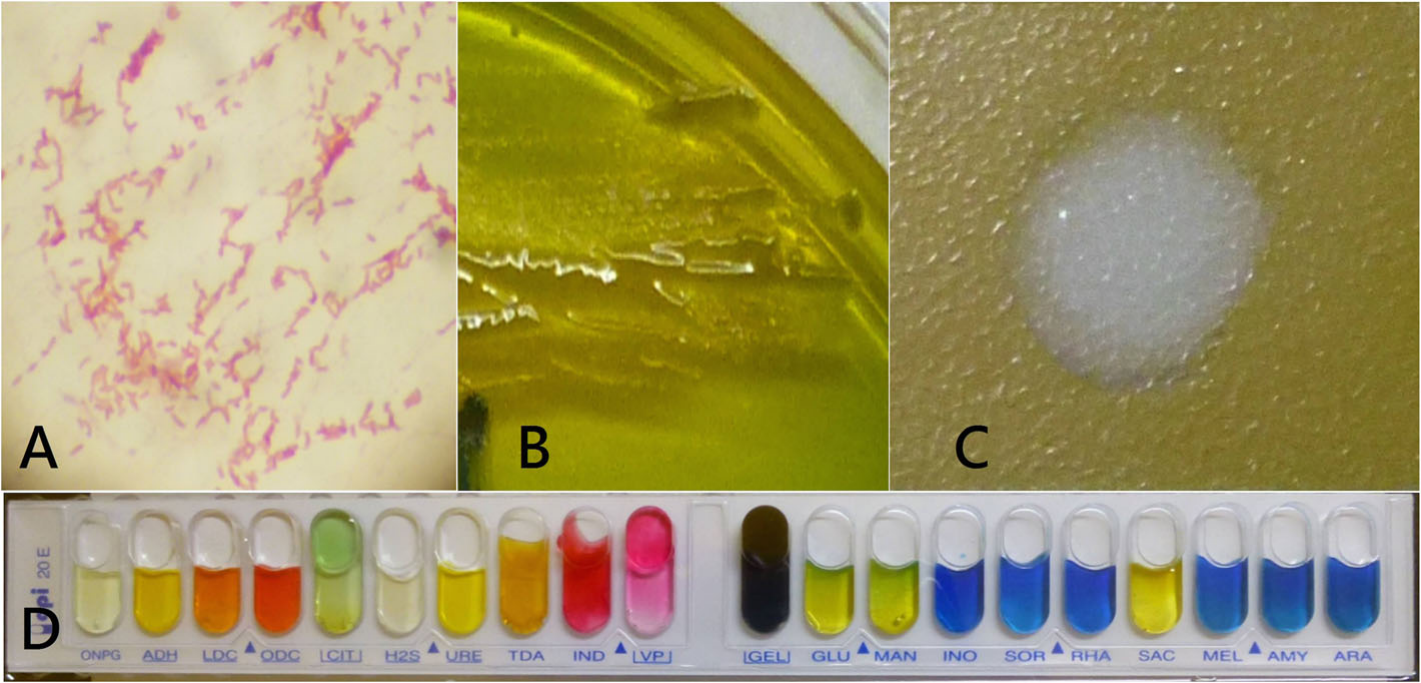
Colonies of *V. cholerae* non-O1 (**a**). Gram stain (**b**). Thiosulfate citrate bile salts sucrose (TCBS) agar (**c**). Negative agglutination test (**d**). The result of Analytical Profile Index 20E (API 20E) Biochemical Test Strip

**Fig. 3:**
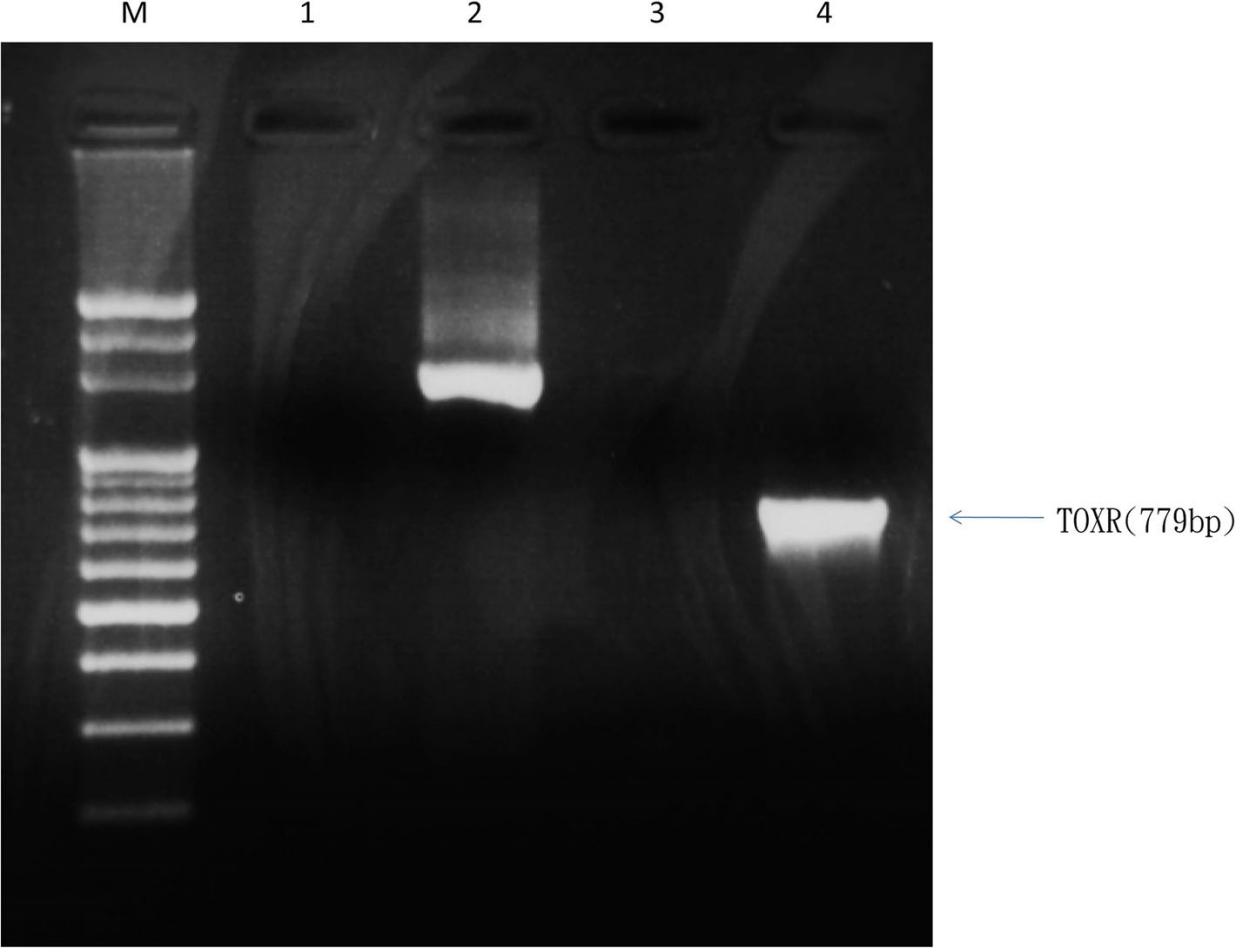
2% Agarose gel electrophoresis files. Lane M: 100-bp DNA ladder; Lane1: d^2^H_2_0 as a Negative control; Lane2: 16S rDNA products amplified by polymerase chain reaction (PCR) using primers 16S1 and 16S2 as a positive control; Lane 3: *Vibrio vulnificus*-specific DNA products amplified in PCR using primers vulrpoS-up and vulrpoS-rp; Lane 4: *V. cholerae* -specific DNA products amplified in PCR using primers F-TOXR and R-TOXR

A 59-year-old healthy fisherman presented with right eye pain, redness, tearing and photophobia for 1 day and the symptoms persisted. According to his statement, his right eye was struck by a marine shrimp while fishing. The patient denied a history of major systemic diseases such as diabetes, hypertension, or liver cirrhosis. The best-corrected visual acuity was 20/60 in the right eye and 20/20 in the left eye. Slit-lamp examination showed moderate conjunctival injection with slight chemosis, and a central stromal infiltration of approximately 1.3 × 1.8mm^2^. Simultaneously, corneal edema with Descemet membrane folding around the lesion was also observed (Fig. 1a). There was no sign of anterior chamber inflammation or endophthalmitis. Initially, under the suspicion of bacterial or fungal infection, the patient was empirically treated with hourly Cravit (levofloxacin oph. Soln, 0.5%, Santen, Japan) and fortified amphotericin B (1 mg/mL). Two days later, right eye pain improved slightly. The gram staining of the corneal culture showed *Vibrio sp.*, and the yellow colony in thiosulfate-citrate-bile salts-sucrose (TBCS) agar was highly identified as *V. cholerae.* Moreover, the negative result in the agglutination test confirmed the non-O1 strain (Fig. 2a-c). Thereafter, the identity of the *V. cholerae* was reconfirmed by laboratory analysis with Analytical Profile Index 20E (API 20E) (Fig. 2d) and polymerase chain reaction (PCR) (Fig. 3)*.* Following the identification of *V. cholerae* non-O1, we halted the use of amphotericin B. Meanwhile, antimicrobial minimal inhibitory concentration testing was performed for this pathogen (Table 1). Based on the results of the test, the antibiotic regimen was changed to topical amikacin (12.5 mg/mL) and Cravit four times daily. One week later, the right uncorrected visual acuity rapidly recovered to 20/25 and corneal infiltration improved (Fig. 1b). The frequency of treatment was tapered gradually without any complication.

**Table 1:** Susceptibilities of the clinical isolate of *V. cholerae* non-O1 to 14 antimicrobial agents

Antimicrobial agent	MIC (ug/ml)
Amikacin	4
Gentamicin	0.5
Tetracycline	1
Tigecycline	0.25
Ciprofloxacin	0.023
Ertapenem	0.023
Imipenem	0.75
Meropenem	0.19
Ceftazidime	0.094
Ceftriaxone	0.016
Cefuroxime	0.125
Ampicillin	1.5
Ampicillin-sulbactam	1
Aztreonam	0.38

*MIC* Minimum Inhibitory Concentration

## Discussion and conclusions

This is the first case of infectious keratitis caused by *V. cholerae* non-O1. Prior to our case, the first published case of ocular infection with *V. cholerae* non-O1 was an endophthalmitis in a cirrhotic patient with septicemia [6]. Corneal pathogenesis of the *V. cholerae* non-O1 strain has not been clearly elucidated yet due to its rare occurrence in ocular tissue. *V. cholerae* non-O1 can potentially bring on pandemic gastroenteritis and extra-intestinal diseases without using lethal virulence factors such as CT and TCP. Some studies have indicated that *V. cholerae* non-O1 possess unique characteristics to evade immune surveillance and destroy its target precisely [7, 8].

Hemolysin, hemagglutinin protease, repeats in toxin (RXT) and type VI secretion system (T6SS) are crucial virulence factors of *V. cholerae* non-O1 [7]. *V. cholerae* non-O1 activates the vacuolating process regulated by hemolysin protein to interfere with intracellular homeostasis and ion exchange, and subsequently impels cellular cytolysis [9]. Therefore, hemolysin-induced vacuolation can potentially disrupt corneal epithelial and endothelial cells to trigger cell damage. Furthermore, occludin and zonula occludens-1 (ZO-1) are enriched in the corneal epithelium as first-line defense components [10]. In *V. cholerae* non-O1, hemagglutinin protease, encoded by the *hap* gene, can degrade essential tight junction-associated protein occludin and disrupt ZO-1 conformation [11]. It is reasonable to infer that *V. cholerae* non-O1 can directly destroy the epithelial occludin and ZO-1 via hemagglutinin protease, digest collagens and abolish corneal transparency. In addition, RTX, akin to exotoxin of *V. cholerae*, was found to be involved in the detachment and round-up of epithelial cells [12]. *V. cholerae* non-O1 can break down corneal epithelium and stroma using the distinctive cytotoxic property of the RTX. T6SS, found in gram-negative bacteria such as *Pseudomonas aeruginosa* and *Escherichia coli*, is a cell-puncturing device that translocates effector proteins into eukaryotic host cells [13]. *P. aeruginosa* has been shown to invades cornea with the assistance of T6SS [14], Hence, it is possible for *V. cholerae* non-O1 to adopt a similar strategy to cause keratitis. These presumptions require further microbiological research and laboratory analysis to determine the pathogenesis of the *V. cholerae* non-O1 keratitis.

In the present case, we initially doubted the presence of this pathogenic strain in the corneal culture because *V. cholerae* has never been reported in keratitis. Hence, it is necessary to validate this pathogen using advanced laboratory methods. The API 20E microtest and real-time PCR are suitable choices. The API 20E is an efficient biochemical kit to discriminate members of gram-negative bacilli in 24 h. Furthermore, the API 20E has a higher detection rate for *V. cholerae* than analogous biochemical panels [15]. On the other hand, real-time PCR enables the most accurate molecular identification for *V. cholera* and is faster than the API 20E detection [16]. Target genes including *hlyA, tcpI, toxR and ompU* are available for the screening of *V. choerae* serotypes [17]. Both of methods are recommended for the diagnosis of intractable infectious ophthalmic due to their advantages of a short reaction time and enhanced accuracy of detection.

*V. cholerae* non-O1 is assumed to be an opportunistic pathogen, although it occasionally causes severe illness [5]. Hence, efficient treatment for *V.* cholerae non-O1 infection has not been determined yet, particularly in ocular diseases. Yang et al. reported the first case of *V. cholerae* non-O1/non-O139 endophthalmitis following septicemia in a cirrhotic patient [6]. This patient received an intravitreal injection of vancomycin and ceftazidime, and a systemic intravenous injection of ceftriaxone (2 g, q12h). Eventually, the patient died of sepsis and respiratory failure. Moreover, sporadic medical regimens for *V. vulnificus* keratitis were delineated. DiGaetano et al. (1989) combined medical treatment and surgical debridement for two cases of *V. vulnificus* keratitis [18]. Massey et al. (2000) used topical therapy with ciprofloxacin and fortified vancomycin for one case of *V. vulnificus* keratitis [19]. Penland et al. (2000) demonstrated that topical application of ciprofloxacin and cefazolin can treat the *V. vulnificus* keratitis [2]. You et al. (2008) used surgical debridement and systemic antibiotics (oral and intravenous injection) for *V. vulnificus* keratitis [20]. Despite the availability of prior case reports (Table 2), therapeutic options for ocular *V. cholerae* non-O1 infection are scant and rudimentary. Our patient with normal immunity completely recovered under topical antibiotic therapy of fluoroquinolone and amikacin. This can be an effective topical regimen for treating *V. cholerae* non-O1 keratitis.

**Table 2:** Reported cases of *Vibrio spp.* Keratitis

Case	Reference	Species	Risk factor	Treatment
1	DiGaetano et al. [18] 1989	*V. vulnificus*	Shucking oysters	Topical: Gentamycin (9.1 mg/mL) Maxitrol^a^ Cefazloin (33 mg/mL) Surgical debridement (day 21)
2	DiGaetano et al. [18] 1989	*V. vulnificus*	Crab shell	Topical: Gentamycin (9.1 mg/mL) Bacitracin (10,000 U/mL) Tetracycline (1%) Tobrmycin (0.3%) Subonjunctival: Gentamycin 20 mg Surgical debridement (day 14)
3	Massey et al. [19] 2000	*V. vulnificus*	Shucking oyster	Topical: Ciprofloxacin Maxitrol^a^ Vancomycin (25 mg/mL)
4	Penland et al. [2] 2000	*V. vulnificus*	Shucking oyster	Topical: Cefazolin (5%) Gentamicin (1.4%) Prednisolone acetate (1%) Ciprofloxacin (0.3%)
5	You et al. [20] 2008	*V. vulnificus*	Wood of boat	Topical: Gatifloxacin Tobramycin Subconjunctival injection Tobramycin (20 mg) Oral: Doxycycline (100 mg/tab) bid Systemic IV: Ceftazidime (2 g) q12h Surgical debridement (day11)
6	Chen et al. 2019	*V. cholerae* non-O1	shrimp	Topical: Levofloxacin (0.5%) Amikacin (12.5 mg/mL)

^a^Maxitrol: Neomycin-polymyxin B

*V. cholerae* non-O1 can cause keratitis after ocular blunt injury by marine creatures and contaminated seawater. A satisfactory outcome can be achieved by comprehensive history taking, early pathogenic diagnosis and compatible antibiotic treatment.

## Data Availability

All data generated or analyzed during this study are included in this published article.

## Abbreviations

API 20E: Analytical Profile Index 20E
CT: Cholera toxin
PCR: Polymerase chain reaction
RXT: Repeats in toxin
T6SS: Type VI secretion system
TBCS: Thiosulfate-citrate-bile salts-sucrose
TCP: Toxin-coregulated pilus
